# Pertactin-Deficient *Bordetella pertussis* with Unusual Mechanism of Pertactin Disruption, Spain, 1986–2018

**DOI:** 10.3201/eid2805.211958

**Published:** 2022-05

**Authors:** Alba Mir-Cros, Albert Moreno-Mingorance, M. Teresa Martín-Gómez, Raquel Abad, Iván Bloise, Magda Campins, Alejandro González-Praetorius, M. Nieves Gutiérrez, Héctor Martín-González, Carmen Muñoz-Almagro, M. Ángeles Orellana, Manuela de Pablos, Josep Roca-Grande, Carlos Rodrigo, M. Elena Rodríguez, Sonia Uriona, M. José Vidal, Tomàs Pumarola, M. Nieves Larrosa, Juan José González-López

**Affiliations:** Hospital Universitari Vall d’Hebron Campus, Barcelona, Spain (A. Mir-Cros, A. Moreno-Mingorance, M.T. Martín-Gómez, M. Campins, H. Martín-González, J. Roca-Grande, S. Uriona, T. Pumarola, M.N. Larrosa, J.J. González-López);; Universitat Autònoma de Barcelona, Barcelona (A. Mir-Cros, A. Moreno-Mingorance, T. Pumarola, M.N. Larrosa, J.J. González-López);; Instituto de Salud Carlos III, Madrid, Spain (R. Abad);; Hospital Universitario La Paz, Madrid (I. Bloise, M. de Pablos);; Hospital Universitario de Guadalajara, Guadalajara, Spain (A. González-Praetorius, M.E. Rodríguez);; Complejo Asistencial Universitario de Salamanca, Salamanca, Spain (M.N. Gutiérrez);; Institut de Recerca Sant Joan de Déu, Esplugues de Llobregat, Spain (C. Muñoz-Almagro);; Universitat Internacional de Catalunya, Barcelona (C. Muñoz-Almagro);; CIBER de Epidemiología y Salud Pública, Barcelona (C. Muñoz-Almagro);; Hospital Universitario 12 de Octubre, Madrid (M.Á. Orellana);; Hospital Universitari Germans Trias i Pujol, Badalona, Spain (C. Rodrigo);; Agència de Salut Pública de Catalunya, Barcelona (M.J. Vidal);; CIBER de Enfermedades Infecciosas, Barcelona (T. Pumarola, M.N. Larrosa, J.J. González-López)

**Keywords:** Bordetella pertussis, whooping cough, pertussis vaccine, phylogenetic evolution, virulence factors, bacteria, Spain

## Abstract

*Bordetella pertussis* not expressing pertactin has increased in countries using acellular pertussis vaccines (ACV). The deficiency is mostly caused by pertactin gene disruption by IS*481*. To assess the effect of the transition from whole-cell vaccine to ACV on the emergence of *B. pertussis* not expressing pertactin in Spain, we studied 342 isolates collected during 1986–2018. We identified 93 pertactin-deficient isolates. All were detected after introduction of ACV and represented 38% of isolates collected during the ACV period; 58.1% belonged to a genetic cluster of isolates carrying the unusual *prn*::del(–292, 1340) mutation. Pertactin inactivation by IS*481* insertion was identified in 23.7% of pertactin-deficient isolates, arising independently multiple times and in different phylogenetic branches. Our findings support the emergence and dissemination of a cluster of *B. pertussis* with an infrequent mechanism of pertactin disruption in Spain, probably resulting from introduction of ACV.

*Bordetella pertussis* is the main causative agent of pertussis, an acute upper respiratory tract infection of humans. The most effective strategy for preventing and controlling this disease is vaccination. In Spain during 1998–2005, pertussis vaccination with whole-cell vaccine (WCV) was progressively replaced by vaccination with acellular pertussis vaccine (ACV), which contains a combination of several antigens. Although vaccines and vaccination programs might differ among countries, the 3-component ACV containing pertussis toxin (PT), filamentous hemagglutinin (FHA), and pertactin is largely used for pertussis vaccination in many countries, including Spain. Specifically, the pertactin component has been included in most vaccines used throughout the history of pertussis vaccination in Spain (Table 1).

**Table 1:** Changes in the pertussis vaccination program in Spain, 1986–2018*

Year	Primary doses				Booster doses		
	Vaccine type	Schedule, mo	Pertussis components		Vaccine type	Schedule	Pertussis components
1975	WCV	3, 5, 7	Inactivated whole cell		NA	No booster	NA
1996	WCV	2–3, 4–5, 6–7	Inactivated whole cell		WCV	15–18 mo	Inactivated whole cell
1998/1999	WCV	2–3, 4–5, 6–7	Inactivated whole cell		ACV	18 mo	PT, FHA, PRN
2001	WCV	2, 4, 6	Inactivated whole cell		ACV	18 mo, 4–6 y	PT, FHA, PRN
2004†	NA	NA	NA		acv	Health workers caring for newborns	PT, FHA, PRN or PT, FHA, PRN, FIM2, FIM3‡
2005/2006	ACV	2, 4, 6	PT, FHA, PRN		ACV	18 mo, 4–6 y	PT, FHA, PRN
2012	ACV	2, 4, 6	PT, FHA, PRN		ACV/acv	18 mo (ACV), 4–6 y (acv)	PT, FHA, PRN or PT, FHA, PRN, FIM2, FIM3‡
2013	ACV	2, 4, 6	PT, FHA, PRN		ACV/acv	18 mo (ACV), 6 y (acv)	PT, FHA, PRN or PT, FHA, PRN, FIM2, FIM3‡
2014/2015§	NA	NA	NA		acv	From 27–28 through 32–36 wk of pregnancy	PT, FHA, PRN or PT, FHA, PRN, FIM2, FIM3‡
2017	ACV	2, 4, 11	PT, FHA, PRN or PT, FHA¶		ACV/acv#	6 y	PT, FHA, PRN or PT, FHA, PRN, FIM2, FIM3‡

*acv, diphtheria–tetanus–acellular pertussis vaccine with reduced antigenic load of diphtheria, tetanus and pertussis; ACV, diphtheria–tetanus–acellular pertussis vaccine; FHA, filamentous haemagglutinin; FIM2, type 2 fimbriae; FIM3, type 3 fimbriae; NA, not applicable; PRN, pertactin; PT, pertussis toxin; WCV, diphtheria–tetanus–whole-cell pertussis vaccine. †Introduction of healthcare worker vaccination. ‡The 5-component ACV, which also contains FIM2 and FIM3, is used in some booster doses. §Introduction of maternal pertussis vaccination. ¶At the end of 2013, use of an ACV not including the PRN component in primary vaccination was approved in Spain. #ACV vaccine is administered to children vaccinated with the 2+1 schedule when they reach 6 y of age. Children vaccinated with the 3+1 schedule receive the acv vaccine.

Despite extensive vaccination campaigns and high vaccination rates, pertussis has resurged in the past 20 years, and outbreaks have occurred worldwide. One of the main causes postulated for the change in pertussis epidemiology is evolution of circulating bacteria to vaccine/immunity-evasive phenotypes (*1*–*4*). In 2007, after the introduction of ACV, pertactin-deficient isolates were detected in France and subsequently in other countries that had adopted ACV (*5*–*9*). Pertactin-deficient strains have demonstrated a greater ability than pertactin-producing strains to colonize ACV-vaccinated animals. Thus, the expansion of pertactin-deficient strains in human populations vaccinated with pertactin-containing vaccines indicates that such strains apparently have a selective advantage in these populations (*10*). The mechanisms associated with loss of pertactin expression are multiple and diverse, including, among others, insertion of the IS*481* and IS*1002* elements in several positions of the pertactin gene, deletions of small parts of or the entire pertactin gene, inversions, and presence of point mutations leading to stop codons (*6*,*11*). Globally, the main factor for pertactin deficiency is still the IS*481* insertion, but other mechanisms are increasing, such as the large inversion in the promotor area and the point mutations in the structural gene (i.e., in positions 223 and 1273 in *prn2*) (*12*–*14*).

To determine the presence of pertactin-deficient *B. pertussis* strains in Spain, we elucidated the genetic mechanisms involved in pertactin loss and bacterial population dynamics, and we analyzed whether replacing WCV with ACV affects emergence of pertactin-deficient *B. pertussis* strains. The study was approved by the Ethics Committee of the Hospital Universitari Vall d’Hebron (reference no. PR(AG)694/2020).

## Methods

### Bacterial Isolates and Study Period

We studied 342 nonduplicate *B. pertussis* clinical isolates collected at 5 hospitals at different locations in Spain during 1986–2018 (Appendix 1). All isolates were obtained from cultures of nasopharyngeal samples collected from patients with pertussis; we excluded isolates collected during studies of contacts. The study period was divided into 3 parts, based on the vaccine type used for routine vaccination in Spain: period 1 (1986–1997; 46/342 isolates) was defined by the exclusive use of WCV; period 2 (1998–2005; 51/342 isolates) was the period of transition to ACV; and period 3 (2006–2018, 245/342 isolates) was when ACV had completely replaced WCV. Isolates were collected from patients with different vaccination status: vaccinated, nonvaccinated, and partially vaccinated (incomplete primary vaccination [1–2 doses] and complete primary vaccination [3–4 doses]).

### Vaccine Antigen Expression

We evaluated production of pertactin, PT, FHA, and fimbrial proteins FIM2 and FIM3. We used an indirect whole-cell ELISA with specific antibodies (97/558 for pertactin, 99/512 for PT S1 subunit, 99/572 for FHA, 06/124 for FIM2, and 06/128 for FIM3; National Institute for Biological Standards and Control, https://www.nibsc.org), as previously described (Appendix 1) (*15*–*17*).

### Whole-Genome Sequencing and Data Analysis

We sequenced all pertactin-deficient isolates detected by ELISA and a proportional random selection of pertactin-producing isolates by using the MiSeq platform (Illumina, https://www.illumina.com) according to a 2 × 300 paired-end protocol. We obtained Bayesian phylogenetic reconstruction with BEAST version 1.10.4 (https://beast.community) by using the general time reversible substitution model, strict clock, and coalescent constant population (Appendix 1). We deposited the genome sequence reads of all 184 *B. pertussis* strains in the National Center for Biotechnology Information database (BioProject no. PRJNA667582) (Appendix 2 Table 1).

## Results

### Temporal Distribution of Pertactin-Deficient *B. pertussis*

**Figure 1:**
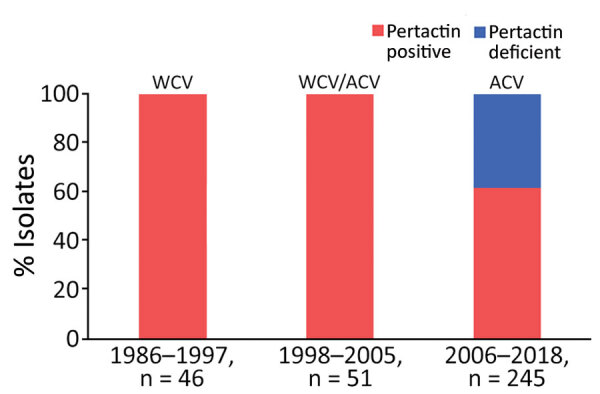
Temporal distribution of pertactin-deficient *Bordetella pertussis* isolates in Spain, 1986–2018. ACV, acellular vaccine WCV, whole-cell vaccine.

**Figure 2:**
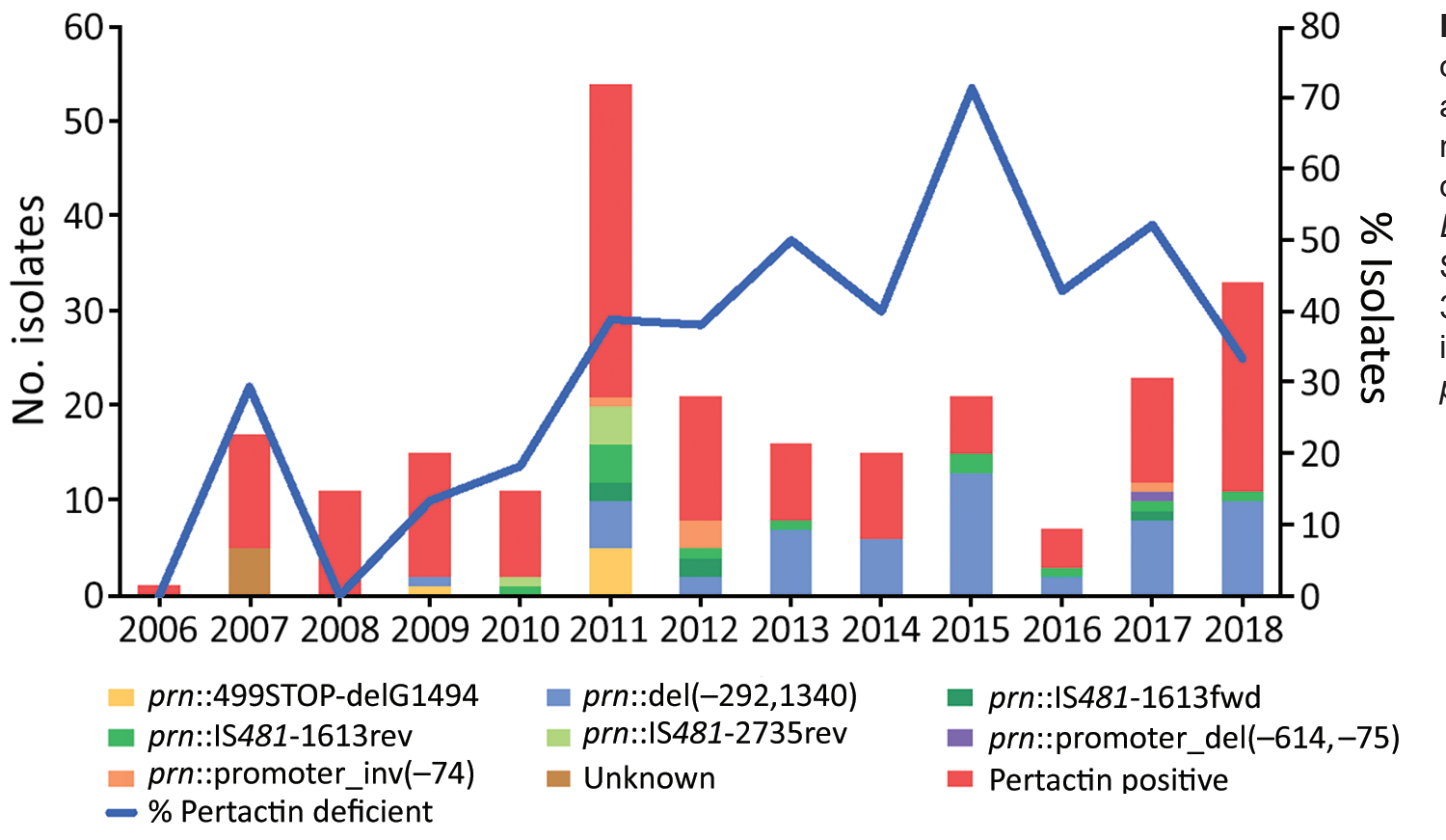
Temporal distribution of pertactin-deficient isolates and temporal trend of molecular mechanisms of pertactin deficiency in pertactin-deficient *Bordetella pertussis* isolates in Spain, 2006–2018 (study period 3). Del, deletion; fwd, forward; inv, inversion; IS, insertion sequence; prn, pertactin gene: rev, reverse.

All pertactin-deficient isolates (93/342) were collected during period 3, representing 38% of the isolates obtained during the period of exclusive ACV administration (Figures 1, 2). The first pertactin-deficient *B. pertussis* isolate was collected in 2007, when prevalence of pertactin-negative *B. pertussis* reached 29.4% of the total isolates collected. Since then, the number of pertactin-deficient isolates progressively increased; prevalence was highest in 2015, the last epidemic year of the disease in Spain, when 71.4% of *B. pertussis* isolates obtained did not express this antigen. Thereafter, prevalence of pertactin-deficient isolates decreased; 33.3% of the isolates collected during 2018 were deficient in production of this antigen. We observed no statistical differences in vaccination status between patients with pertactin-deficient and pertactin-producing *B. pertussis* infections (χ^2^ test, p>0.05; Appendix 2 Table 1).

### Molecular Mechanisms of Pertactin Deficiency

We identified 7 mechanisms involved with pertactin deficiency (Table 2; Appendix 2 Table 1). Among these mechanisms, we found partial deletion of the promoter zone and part of the pertactin-encoding gene located between positions –292 and 1340 (*prn*::del(–292, 1340)) in 54 (58.1%) of the 93 pertactin-deficient isolates. This mutation had been observed in 1 isolate collected in 2009 and was the most detected mechanism of pertactin deficiency since 2011, except for 2012 (Table 2; Figure 2). The second most common mechanism of pertactin deficiency was the IS*481* insertion at position 1613–1614 in reverse orientation (*prn*::IS*481*-1613rev). This mutation was identified in 12 (12.9%) of the pertactin-deficient isolates; it was identified in 2010 and remained as a mechanism of pertactin deficiency over the following years, except for 2014, when it was not found in any of the isolates collected (Table 2; Figure 2). Occasionally, we identified other minor causes of pertactin deficiency (Table 2; Figure 2; Appendix 2 Table 1). It was not possible to identify the genetic mechanism underlying the pertactin deficiency in 5 (5.4%) of the pertactin-deficient isolates collected during 2007 because no mutation was identified in the pertactin promoter or the structural gene (Figure 2; Appendix 2 Table 1).

**Table 2:** Genomic mechanisms leading to origin deficiency of pertactin among *Bordetalla pertussis* isolates in Spain, 2006–2018*

Mechanism type	Mechanism name	Mechanism description	Genomic location†	Isolates, no. (%)	Reference
Deletion	*prn*::499STOP-delG1494	G deletion	1494	6 (6.5)	This study
	*prn*::del(–292, 1340)	Within promoter and first part of *prn* gene	–292 to 1340	54 (58.1)	(*18*)
	*prn*::promoter_del(–614, –75)	Within promoter	–614 to –75	1 (1.1)	This study
Insertion	*prn*::IS*481*-1613fwd	IS*481* within *prn*, forward direction	1613–1614	5 (5.4)	(*6*)
	*prn*::IS*481*-1613rev	IS*481* within *prn*, reverse direction	1613–1614	12 (12.9)	(*6*)
	*prn*::IS*481*-2735rev	IS*481* within *prn*, reverse direction	2735–2736	5 (5.4)	(*6*,*19*)
Inversion	*prn*::promoter_inv(-74)	22 kb large inversion within promoter	–20892 to –75	5 (5.4)	(*6*)

*del, deletion; fwd, forward insertion; inv, inversion; IS, insertion element; *prn*, pertactin gene; rev, reverse insertion. †Numbers indicate the position of each mechanism relative to the *prn2* start codon.

### Phylogenetic Analysis

**Figure 3:**
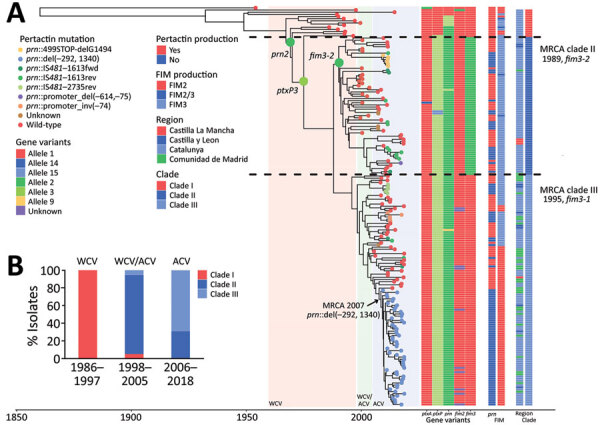
Time-scaled phylogeny of *Bordetella pertussis* isolates collected in Spain, 1986–2018. A) Bayesian phylogenetic reconstruction of 184 *B. pertussis* isolates and the reference Tohama I (GenBank accession no. NZ_CP031787). Shaded regions indicate periods of WCV, WCV/ACV, and ACV use. Colored dots at the end of the tree branches indicate pertactin production for each isolate. Alleles of *ptxA*, *ptxP*, *prn*, *fim2*, and *fim3* are indicated for each isolate, on the right. Data associated with expression (serotyping) of FIM2/FIM3 are also indicated for each isolate; B) Temporal distribution of the isolates’ clades of *B. pertussis* based on the vaccine type(s) used for routine vaccination. ACV, acellular vaccine; del, deletion; FIM, fimbrial serotype; fwd, forward; inv, inversion; IS, insertion sequence; MRCA, most recent common ancestor; prn, pertactin gene: rev, reverse; WCV, whole-cell vaccine.

To gain insight into the pertactin-deficient *B. pertussis* population dynamics in Spain, we reconstructed Bayesian phylogeny with a selection of 184 isolates: the 93 pertactin-deficient isolates and 91 pertactin-producing isolates randomly collected over the entire period. Whole-genome sequence variation analysis identified 1,255 single-nucleotide polymorphisms (SNPs). Bayesian evolution analysis conducted with BEAST estimated the mean evolutionary rate of *B. pertussis* as 2.7 × 10^–7^ substitutions/site/year (95% highest posterior density [HPD] 2.4–3 × 10^–7^ substitutions/site/year), corresponding to 1.1 substitutions/genome/year. Bayesian model comparison confirmed that the general time reversible substitution model, strict clock, and coalescent constant population were the best fitting for the alignment. According to the type 3 fimbriae allele and the genetic identity of the isolates, we defined 3 clades within the phylogenetic tree (Figure 3, panel A; Appendix 2 Table 1). We found a strong association between the period and the clades of the circulating isolates (Figure 3, panel B). All isolates belonging to clades I, II, and III were producers of PT and FHA, regardless of pertactin loss (Appendix 2 Table 1).

Clade I, which was the most predominant clade during the exclusive WCV period, included 13 isolates obtained during 1986–1999 and 1 isolate collected in 2014. Overall, 84.6% contained the *ptxA1/ptxP1/fim2-1/fim3-1* allelic combination; for pertactin, 38.5% encoded *prn2*, 38.5% *prn3*, and 23.1% *prn1*. With regard to fimbrial serotypes, 46.2% were FIM3, 38.5% FIM2, and 15.4% FIM2/3. No pertactin-deficient isolates were observed among the isolates belonging to this clade (Figure 3; Appendix 2 Table 1).

Clade II, which predominated during the period of transition from WCV to ACV, included 64 isolates collected during 1998–2018, of which 93.8% contained the *ptxA1/ptxP3/prn2/fim2-1/fim3-2* allelic combination (Figure 3; Appendix 2 Table 1). With regard to fimbrial serotypes, 96.9% expressed FIM3 and 3.1% expressed both types of fimbriae simultaneously (FIM2/3). BEAST analysis estimated the time to the most recent common ancestor of the clade II isolates to be 1989 (95% HPD 1987–1992). With regard to pertactin production, 24 (37.5%) of the isolates from this clade were pertactin deficient. Of these, 13 (54.2%) contained a mutation associated with any of the IS*481* insertions described at position 1613–1614, distributed in different branches within the clade. Among these isolates, we identified a cluster of 6 isolates possessing the *prn*::IS*481*-1613rev mutation. The isolates were obtained in Barcelona during 2011–2017 (range 1–11 SNPs). In addition, 6 (25%) of the pertactin-deficient isolates within clade II shared the *prn*::499STOP-delG1494 mutation; all were genetically closely related, as they clustered together (range 0–4 SNPs). Samples containing these isolates were collected in Barcelona, and all but 1 was obtained during March–September 2011. No epidemiologic link was identified among the patients from whom these isolates were obtained. One pertactin-deficient isolate found in clade II showed the *prn*::promoter_del(–614, –75) mutation. All pertactin-deficient isolates from this clade, including *prn*::IS*481*-1613, *prn*::499STOP-delG1494 and *prn*::promoter_del(–614, –75) mutations, presented the FIM3 serotype (Figure 3; Appendix 2 Table 1).

**Figure 4:**
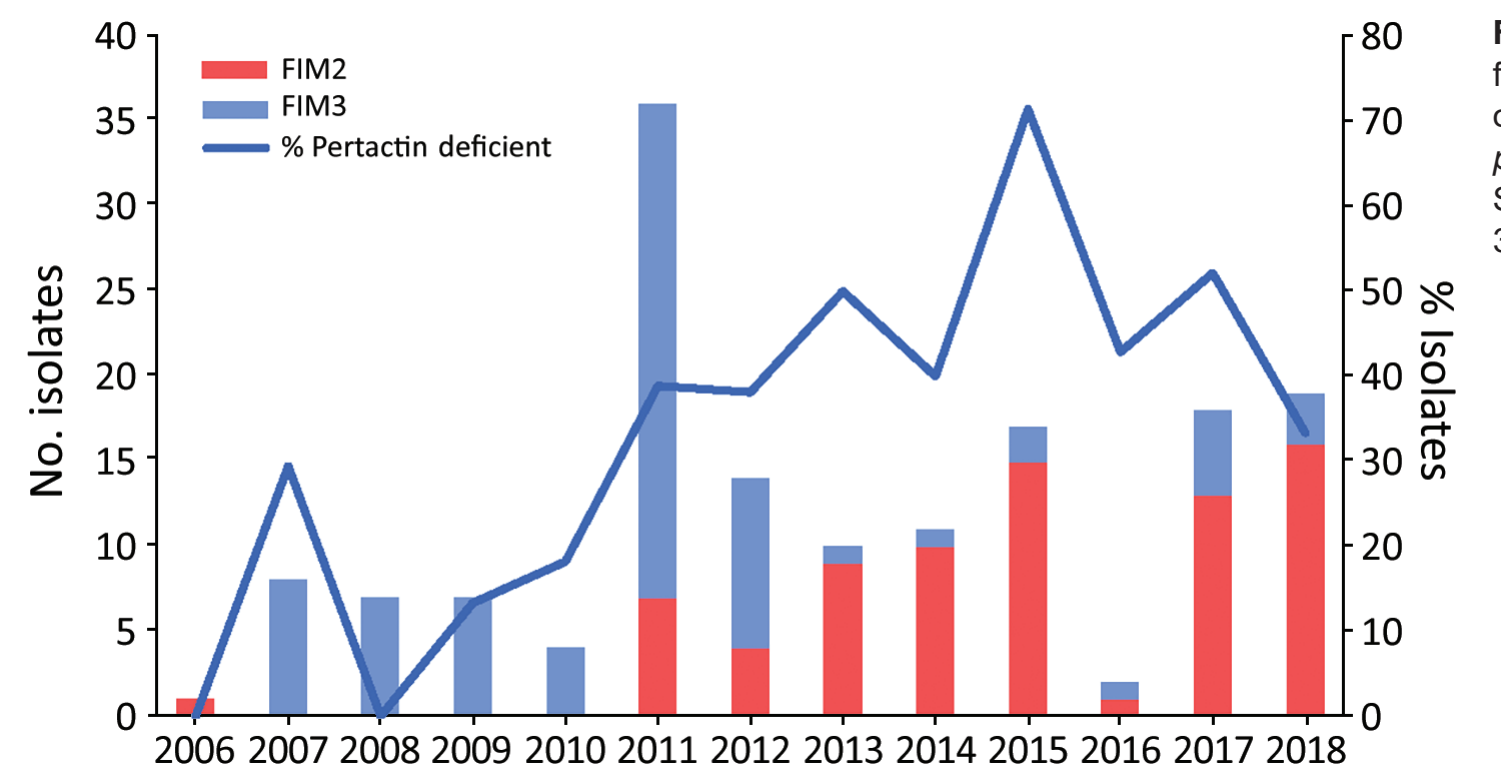
Temporal distribution of fimbrial serotypes and frequency of pertactin-deficient *Bordetella pertussis* isolates collected in Spain, 2006–2018 (study period 3). FIM, fimbrial serotype.

Clade III, which was the most predominant during the exclusive ACV period, consisted of 107 isolates collected during 2005–2018, of which 89.7% possessed the *ptxA1/ptxP3/prn2/fim2-1/fim3-1* allelic combination (Figure 3; Appendix 2 Table 1). With regard to fimbrial serotype, 72% of isolates of this clade expressed FIM2 and 28% FIM3, observed as FIM2 isolates replaced the previously predominant fimbrial serotype FIM3 from 2013 and coinciding with the incremental detection of pertactin-deficient isolates (Figure 4; Appendix 2 Table 1). BEAST analysis identified the time to the most recent common ancestor of clade III isolates as 1995 (95% HPD 1992–1998). Regarding pertactin production, 69 (64.5%) of the isolates of this clade were pertactin deficient. Of these, 54 (78.3%) possessed the *prn*::del(–292, 1340) mutation, forming a large cluster of isolates (range 0–19 SNPs of difference among them) obtained during 2009–2018 in Barcelona, Madrid, and Salamanca, Spain (estimated divergence occurring in 2007 [95% HPD 2005–2008]). Two other minor clusters of pertactin-deficient isolates with the same mechanism of pertactin deficiency were identified in clade III. The first cluster included 5 (7.2%) of the pertactin-deficient isolates within the clade (range 0–5 SNPs); all shared the *prn*::IS*481*-2735rev mutation and were collected during 2010–2011 in Barcelona, Madrid, and Salamanca. The second cluster also included 5 (7.2%) of the pertactin-deficient isolates within the clade (range 3–20 SNPs), possessed the *prn*::promoter_inv(–74) mutation, and was obtained during 2011–2017 in Barcelona, Madrid, and Salamanca. Of this clade, 4 (5.8%) pertactin-deficient isolates possessed a mutation associated with any of the described insertions of IS*481* at position 1613–1614; all were distributed randomly at different branches along the clade. Combining the deficiency of pertactin and the fimbrial serotype, *prn*::del(–292, 1340) isolates were associated with FIM2 expression, whereas *prn*::IS*481*-2735rev and *prn*::promoter_inv(–74) isolates were related to the FIM3 serotype. Last, 50% of pertactin-deficient isolates of clade III with the *prn*::IS*481*-1613 mutation expressed FIM2 and 50% possessed the FIM3 serotype (Figure 3; Appendix 2 Table 1).

We performed an SNP analysis to identify the genetic relationship among pertactin-deficient isolates showing clonal expansion in this study (i.e., *prn*::promoter_inv(–74), *prn*::del(–292, 1340), and *prn*::IS*481*-2735rev) and isolates that possessed the same pertactin gene mutation but were identified in other countries (i.e., Australia, France, and the United States) (Appendix 2 Table 2). The analysis revealed that isolates with the same pertactin gene–disruption mechanism nested together independently of the country in which they were obtained (Appendix 1 Figure).

## Discussion

Pertactin-deficient isolates have been reported in several countries with a history of widespread vaccination with ACV containing pertactin. Nonetheless, there are no data for the pertactin-deficient *B. pertussis* strains in Spain. We detected pertactin-deficient isolates in Spain emerging concurrently with the introduction of ACV. We found pertactin-deficient isolate prevalence to be 38% during 2006–2018, the period of exclusive ACV use in this country.

In Spain, booster vaccination with ACV was introduced in the late 1990s and primary vaccination with ACV was begun in 2005. In 2007, shortly after implementation of ACV as the only vaccine administered against pertussis, the first pertactin-deficient isolate of this study was identified. After that, prevalence of pertactin-deficient *B. pertussis* progressively increased, reaching the highest prevalence (71.4%) in 2015. These results suggest that ACV use has probably driven an antigenic shift of *B. pertussis* toward loss of pertactin expression. This finding is in line with previously reported findings from several other countries, supporting the hypothesis that emergence of pertactin-deficient isolates depends on time since introduction of ACV containing pertactin (*6*,*13*). In a multicenter study conducted in Europe, in which ACV was introduced in several countries at the end of the 1990s, the proportion of pertactin-deficient isolates identified during 2007–2009 was 6.4% and during 2012–2015, the proportion increased to 24.9% (*13*). In Japan, ACV was first introduced in 1981 and 41% of pertactin-deficient isolates were detected during 2005–2007 (*20*). Similarly, in the United States, where ACV was introduced in 1991, 85% of *B. pertussis* isolates collected during 2011–2013 were pertactin deficient; and in Australia, the proportion of pertactin-deficient isolates reached 78% in 2012, after introduction of ACV in 1997 (*8*,*14*,*21*). However, a recent study conducted in Japan revealed a surprising decrease (to <10%) in prevalence of pertactin-deficient *B. pertussis* during 2014–2016. Furthermore, a genotypic replacement from the *ptxA2*/*ptxP1*/*prn1* to the *ptxA1*/*ptxP3*/*prn2* profile coinciding with the decline in pertactin-deficient *B. pertussis* was observed (*7*,*20*,*22*,*23*). The most likely explanation was the effect of the 2012 introduction of ACV not including the pertactin component (*20*). In our study, a decrease in pertactin-deficient isolates within the *B. pertussis* population has been observed since 2016. No changes have been detected in vaccination coverage in Spain (>95% of coverage in primary vaccination since 1999) (*24*). However, introduction of a vaccine without the pertactin component used for primary vaccination was approved in Spain in 2013 and has been administered in 5 of 19 regions (not in Catalunya or the Comunidad de Madrid). Given that its use in Spain remains limited, establishing a possible causal relationship between its introduction and the loss of selective pressure towards pertactin-deficient isolates is difficult. Another factor that might have contributed to the increase of pertactin-producing isolates could be the progressive increase of the population with no immunity against pertactin as a consequence of natural immunization after infection by pertactin-deficient isolates over recent years. Nonetheless, in countries in which monocomponent vaccines (including PT only) or WCV were used, pertactin-deficient isolates were also observed. Examples include Denmark and Poland, where 14.8% of isolates collected during 2012–2015 and 15.4% of isolates collected during 2010–2016 were pertactin deficient (*13*,*25*). Intercountry circulation of pertactin-deficient strains among neighboring countries in which the ACV vaccine is used could explain dissemination of these isolates in these countries. To the contrary, few or no pertactin-deficient isolates were detected in countries such as Iran or Argentina, where WCV is used for primary vaccination, because there may be less selection pressure and less advantage for pertactin-deficient strains to emerge in a WCV-immunized population (*26*,*27*). Overall, these observations would support the hypothesis that pertactin-deficient isolates are selected in response to host immunity against pertactin after vaccination with ACV that contains this antigen (*20*).

Emergence of pertactin-deficient *B. pertussis* isolates in Spain has not resulted from an event of clonal emergence and dissemination because no single common ancestor has been found for all these isolates. We found that diverse mechanisms of pertactin gene disruption originated in different lineages distributed throughout the phylogeny of *B. pertussis*. This same phenomenon has been described in the United States, Japan, Australia, and several countries in Europe (*6*,*14*,*22*,*28*). The most commonly observed mechanism of disruption in our study (58.1%) was the unusual *prn*::del(–292, 1340), which implies a deletion of ≈1.6 kb. The second most commonly observed mechanism of pertactin deficiency (23.7%) was the IS*481* insertion at different locations along the pertactin gene (at positions 1613–1614 and 2735–2736, in either forward or reverse orientation). To the contrary, this mechanism has been the most frequently detected mechanism for pertactin deficiency in studies performed in other countries, such as Australia, Europe, and the United States, where 88.6%, 48.5% and 47.4% of the *B. pertussis* collected showed IS*481* as the main mechanism involved in pertactin gene disruption (*13*,*14*,*28*). Surprisingly, although *prn*::del(–292, 1340) has been identified sporadically in isolates from other countries, as far as we know, it has not been detected as a major mechanism of pertactin deficiency as observed in this collection of *B. pertussis* isolates in Spain. Specifically, this deletion has recently been detected in the United States in 1 isolate obtained in 2016 (*18*). It has also been detected in 5.9% of the pertactin-deficient isolates in Slovenia in a study conducted during 2006–2017, in 2.9% in Australia in a study conducted during 2013–2017, and in 5.6% of the recent pertactin-deficient isolates detected in France (*12*,*28*,*29*). All these isolates had the *ptxA1*/*ptxP3*/*fim3.1* genotype as observed in the isolates identified in our study (no data available for isolates from France) (*14*,*28*,*29*). As previously stated, vaccination coverage has not changed over the past 2 decades and no other demographic or epidemiologic factors that might have been involved in the successful dissemination of *prn*::del(–291, 1340) isolates in Spain have been identified. In addition, these isolates clustered together in the *B. pertussis* phylogeny and arose within the period of ACV use in Spain, suggesting that ACV implementation might have contributed to emergence of isolates containing this mutation and their dissemination in the environment. That the high prevalence observed is not the result of an event of outbreak-related dissemination is suggested by the genetic distance they possess; the fact that they have been found in different regions of Spain in different years; and the finding that isolates from Australia, France, and the United States possess this mutation also grouped in a monophyletic cluster with the isolates from our study (range 0–19 SNPs). Therefore, expansion of these isolates in Spain but not in other countries could be interpreted as successful intracountry dissemination of this lineage of isolates. Continued monitoring of their prevalence and evolution, especially among neighboring countries, is needed.

Our findings revealed that the fimbrial serotype of the *B. pertussis* circulating strains has shifted over the years, although most of the ACV used does not contain FIM antigens because FIM2 isolates replaced the previously predominant fimbrial serotype FIM3 from 2013, coinciding with increased detection of pertactin-deficient isolates. Although information regarding the type of ACV vaccine administered is not available, fimbrial serotype replacement in *B. pertussis* is common and has been previously associated with immunity induced by vaccine or natural infection (*30*,*31*). When comparing the fimbrial serotype with pertactin deficiency, we observed that pertactin-deficient isolates collected at the beginning of the ACV period (2007–2012) were mostly associated with FIM3. Likewise, pertactin-deficient isolates collected in the last years of the ACV period (2013–2018) were mainly associated with FIM2, concurring with the shift in fimbrial serotype observed in the circulating *B. pertussis* population. Similarly, in other studies conducted in Europe, 84.9% of pertactin-deficient isolates obtained during 1998–2015 were FIM3, whereas all pertactin-deficient isolates collected in Slovenia during 2014–2017 were FIM2, denoting fimbrial serotype replacement as a consequence of immunity induced by the previous circulation of FIM3 isolates (*13*,*29*). In our study, the fimbrial serotype shift toward FIM2 mostly likely resulted from emergence of isolates with the *prn*::del(–292, 1340) mutation, suggesting a possible link between the 2 characteristics, which could provide an adaptive advantage of the isolates to escape population immunity, whether generated by vaccination or by natural infection by FIM3-producing *B. pertussis*.

One study limitation might be underdetection of low-prevalence *prn* mutations as a consequence of the number of isolates included per year, the overrepresentation of isolates from the ACV period, and the higher number of isolates collected from the Catalunya region. However, we provide a representative view of the mutations that have conditioned the emergence of pertactin-deficient *B. pertussis* in Spain because we did not include isolates from contacts of case-patients and the most prevalent pertactin-deficiency mechanisms found were detected in different regions of Spain in different years.

Our results show how introduction of ACV concurred with emergence of pertactin-deficient *B. pertusiss* in Spain. Several mechanisms are responsible for this phenomenon; the most identified mutation is *prn*::del(–292, 1340), found in a specific cluster of *B. pertussis*, which emerged after the implementation of vaccination with ACV. This finding is contrary to what has been observed in other countries, in which an IS*481-*mediated pertactin gene disruption has been the main mechanism identified. Other factors may have contributed to the dissemination of pertactin-deficient isolates in Spain, reinforcing the value of long-term surveillance of *B. pertussis* populations and their antigenic characteristics to assess the role that different pathogen adaptation mechanisms may have in the emergence of pertussis.

Appendix 1 Supplemental methods and results for study of pertactin-deficient *Bordetella pertussis* with unusual mechanism of pertactin disruption, Spain, 1986–2018.

Appendix 2 Detailed data about the *Bordetella pertussis* isolates included in the phylogenetic analysis of pertactin-deficient *B. pertussis* with unusual mechanism of pertactin disruption, Spain, 1986–2018.
