# Supplementary material for: Pertactin-Deficient Bordetella pertussis with Unusual Mechanism of Pertactin Disruption, Spain, 1986–2018
Source: Emerg Infect Dis. 2022 May;28(5):967–76. doi: 10.3201/eid2805.211958 (PMC9045434; doi:10.3201/eid2805.211958)
Supplement: Appendix 1 — Supplemental methods and results for study of pertactin-deficient Bordetella pertussis with unusual mechanism of pertactin disruption, Spain, 1986–2018. [file 21-1958-Techapp-s1.pdf]

Article DOI: https://doi.org/10.3201/eid2805.211958

Pertactin-Deficient *Bordetella pertussis* with Unusual Mechanism of Pertactin Disruption, Spain, 1986–2018

Appendix 1

Detailed Methods

Bacterial isolates and study period

Among the 342 *B. pertussis* clinical isolates obtained during 1986–2018, 260 isolates were collected at the Hospital Universitari Vall d'Hebron (Barcelona, Catalunya), 26 isolates were collected at the Hospital Universitario 12 de Octubre (Madrid, Comunidad de Madrid), 25 isolates were collected at the Hospital Universitario La Paz (Madrid, Comunidad de Madrid), 16 isolates were collected at the Hospital Universitario de Salamanca (Salamanca, Castilla y León) and 15 isolates were collected at the Hospital Universitario de Guadalajara (Guadalajara, Castilla La Mancha).

A comprehensive review of patients' medical records was performed to collect the possible epidemiological links among the pertussis cases. In the case of an educational center or familiar outbreak, only one representative isolate was included in the study.

Vaccine antigen expression

The production of PRN was performed in all isolates collected during 1986–2018 (n=342) whereas, the production of PT and FHA and serotyping were conducted with the selected isolates included in the phylogenetic analysis (n=184). For indirect whole-cell enzyme-linked immunosorbent assay (ELISA), 100 µl of inactivated bacterial suspension (0.1 at 620 nm) was coated overnight at room temperature in polystyrene microtiter plates. The next day, normal serum (R9133-rabbit or S3772-sheep, Sigma-Aldrich) with PBS at 1:100 was used to block the plate. For the detection of *B. pertussis* antigen-production, specific antibodies to PRN, PT, FHA, FIM2 and FIM3 (97/558, 99/512, 99/572, 06/124 and 06/128, respectively, NIBSC, https://www.nibsc.org) produced in sheep or mice was used (diluted in Normal Serum-PBS,

according to the manufacturer's conditions). Alkaline-phosphatase-conjugated (Anti-sheep/mouse IgG-AP, Sigma-Aldrich) diluted 1:10,000 with normal serum-PBS was used as a secondary antibody. Finally, alkaline-phosphatase substrate (S0942, Sigma-Aldrich) was added to the plate. The absorbance was measured at 405 nm after 1 hour of incubation at 37°C and the addition of stop buffer (NaOH 3M). One PRN-deficient isolate collected in France was used (FR3640) as a negative control, and the reference strains Tohama I (FIM2), B1900 (FIM3) and B3313 (FIM2 and FIM3) were incorporated as positive controls.

Whole genome sequencing and data analysis

For whole genome sequencing, DNA libraries were constructed using the Nextera DNA Flex Library Prep Kit (Illumina, https://www.illumina.com) prior to sequencing with the MiSeq System (Illumina, San Diego), with a 2x300 paired-end protocol. Paired-end reads were trimmed and assembled using Trimmomatic version 0.39 (http://www.usadellab.org/cms/?page=trimmomatic) and Velvet version 2.0 (https://www.ebi.ac.uk/~zerbino/velvet/), respectively. The identification of mutations in the PRN gene was performed from *de novo* assembled genomes through the CLC Genomics Workbench program and the BLAST tool version 2.10.1 (https://blast.ncbi.nlm.nih.gov/Blast.cgi) using *Bordetella pertussis* Tohama I (GenBank accession no. NZ_CP031787) as query and J625 (NZ_CP022362, *prn*::del(-292, 1340)), I182 (NZ_CP026996, *prn*::IS*481*-1613rev), H911 (NZ_CP011238, *prn*::IS*481*-1613fwd), J162 (NZ_CP013899, *prn*::IS*481*-2735rev) and H920 (KF804027, *prn*::promoter_inv(-74)) for confirmation. The presence of mutations other than IS*481* insertion were confirmed mapping the trimmed reads and inspecting the bam file with IQV (version 2.11.2) against the genome of *Bordetella pertussis* Tohama I (GenBank accession no. NZ_CP031787). Trimmed reads were used for detecting SNP variants with Snippy version 4.3.6 (https://github.com/tseemann/snippy), using *Bordetella pertussis* Tohama I as a reference strain (GenBank accession no. NZ_CP031787). A maximum likelihood analysis was performed with IQTree version 1.6.10 (http://www.iqtree.org) using 1,000 bootstrap replicates. The temporal signal was analyzed by plotting root-to-tip divergence versus sampling time of the maximum likelihood tree with TempEst version 1.5.3 (http://tree.bio.ed.ac.uk/software/tempest).

Bayesian Evolutionary Analysis Sampling Trees (BEAST version 1.10.4, https://beast.community) was used to infer the phylogenetic dynamics. The general time-

reversible model (GTR) of nucleotide substitution was used, which was selected as best fitting in the bModelTest version 1.2.1 (https://github.com/BEAST2-Dev/bModelTest). Different clock models and coalescent priors were compared with path sampling with 100M chain, and strict clock and uncorrelated log normal clock were tested as well as a coalescent constant population, coalescent exponential population, and Bayesian Skyline. As a result, a strict clock and constant population was used to infer the temporal scale of the evolutionary process.

In order to determine the phylogenetic relation of the isolates presenting the *prn*::promoter_inv(-74), *prn*::del(-292, 1340) and *prn*::IS*481*-2735rev mutation and other isolates presenting these mutations are available in public databases (Appendix 2 Table 2, https://wwwnc.cdc.gov/EID/article/28/5/21-1958-App2.xlsx). A maximum likelihood analysis was performed as explained above.

Statistical analysis

Differences between vaccinated, partially vaccinated (complete primary vaccination and incomplete primary vaccination) and unvaccinated patients and PRN-production, categorical variables were compared using the χ^2 test. Two tail p values <0.05 were considered significant.

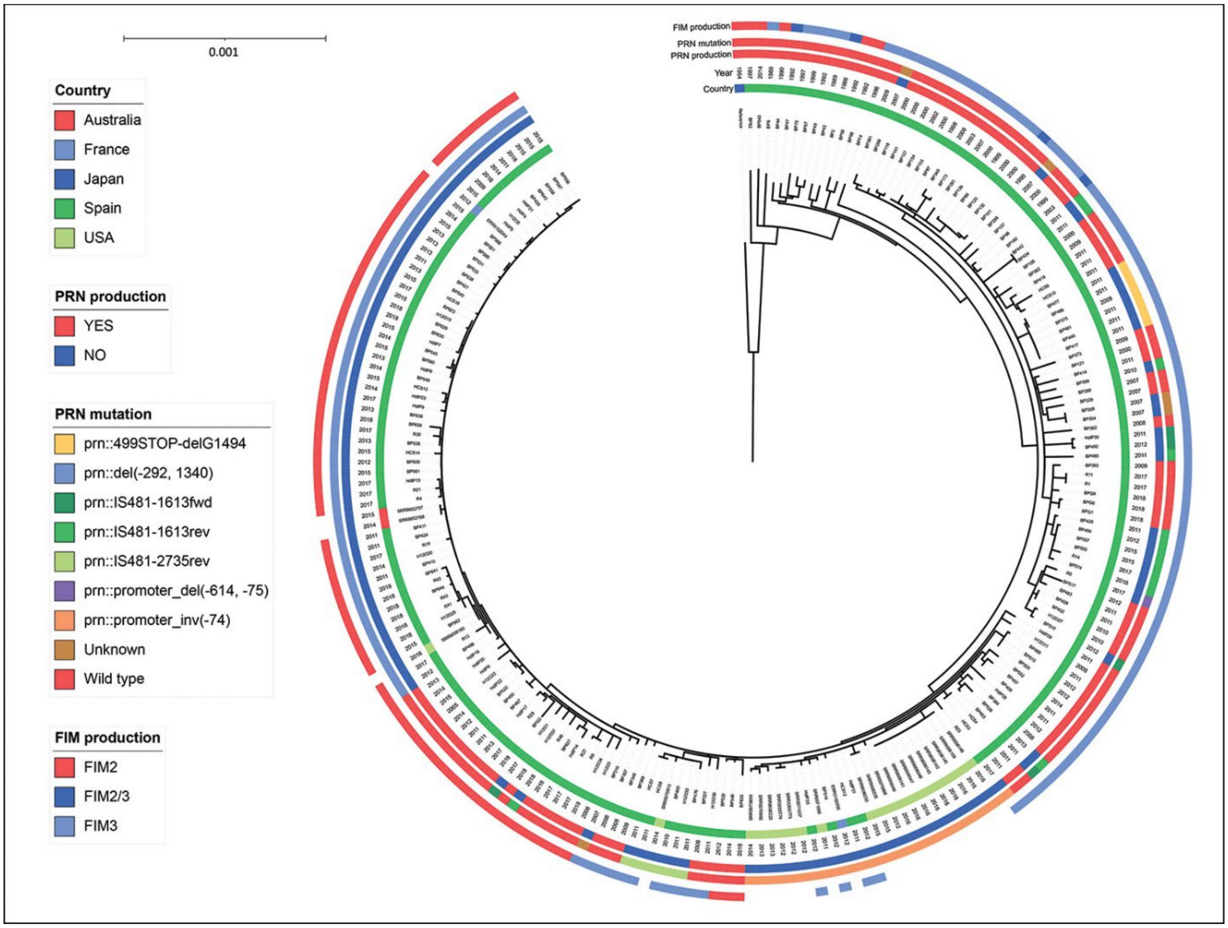

Appendix Figure. Maximum likelihood tree of the 184 *B. pertussis* isolates and 25 PRN-deficient international selected *B. pertussis* isolates. The tree was rooted with the reference *B. pertussis* Tohama I (GenBank accession no. NZ_CP031787). The characteristics of the isolates, including collection country and year, PRN production and PRN mutation and FIM production, are color coded, as detailed in the key. The scale bar indicates substitutions per site. PRN, pertactin; prn, pertactin gene; del, deletion; IS, insertion element; fwd, forward insertion; rev, reverse insertion; inv, inversion; FIM, fimbrial serotype.
